# Supplementary material for: Disulfide cross-linked redox-sensitive peptide condensates are efficient cell delivery vehicles of molecular cargo
Source: bioRxiv. 2025 May 23:2025.05.20.655132. Preprint. [Version 1] doi: 10.1101/2025.05.20.655132 (PMC12139793; doi:10.1101/2025.05.20.655132)
Supplement: 1 [file NIHPP2025.05.20.655132V1-supplement-1.pdf]

bioRxiv preprint doi: https://doi.org/10.1101/2025.05.20.655132; this version posted May 23, 2025. The copyright holder for this preprint (which was not certified by peer review) is the author/funder, who has granted bioRxiv a license to display the preprint in perpetuity. It is made available under aCC-BY 4.0 International license.

SUPPLEMENTARY FIGURES

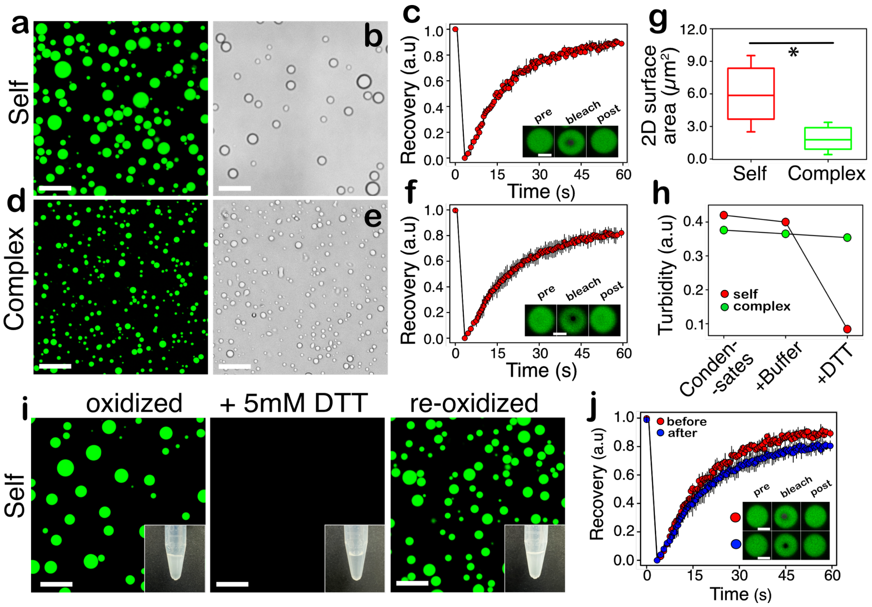

Figure S1. Characterization of PSP-2 condensates in buffer. (a) Confocal microscopy and (b) brightfield images of 3.5 mM PSP-2 peptide self-coacervates in 50 mM tris buffer with 2.5 M NaCl, pH 8.0 at 37 °C. (c) Confocal microscopy and (d) brightfield images of complex-coacervates containing 200 μM PSP-2 with 200 μg/mL PolyA-RNA in the same buffer but with 50 mM NaCl. For confocal visualization of the droplets, 1% FITC-labeled peptide was added to the reactions. (e, f) Fluorescence recovery after photobleaching (FRAP) analysis of condensates formed by self- and complex-coacervation, respectively, along with the regions of interest (ROIs; insets). (g) Sizes of the droplets were quantified based on the 2D surface area observed in the images using ImageJ software. * $p<0.05$. (h) The dissolution of condensates was demonstrated by turbidity; the reducing agent, DTT, was added to a final concentration of 5 mM. An identical volume of buffer without DTT was added as a negative control. (i) Confocal microscopy images of FITC-labeled PSP-2 peptide self-coacervates under respective LLPS conditions. The air-oxidized condensates were first dissolved using DTT and re-oxidized using hydrogen peroxide. The insets show Eppendorf tubes in their respective conditions. (j) FRAP analysis to assess the

bioRxiv preprint doi: https://doi.org/10.1101/2025.05.20.655132; this version posted May 23, 2025. The copyright holder for this preprint (which was not certified by peer review) is the author/funder, who has granted bioRxiv a license to display the preprint in perpetuity. It is made available under aCC-BY 4.0 International license.

viscosity of the self-coacervates before and after reoxidation along with ROIs in the insets. The scale bar is 20 µm.

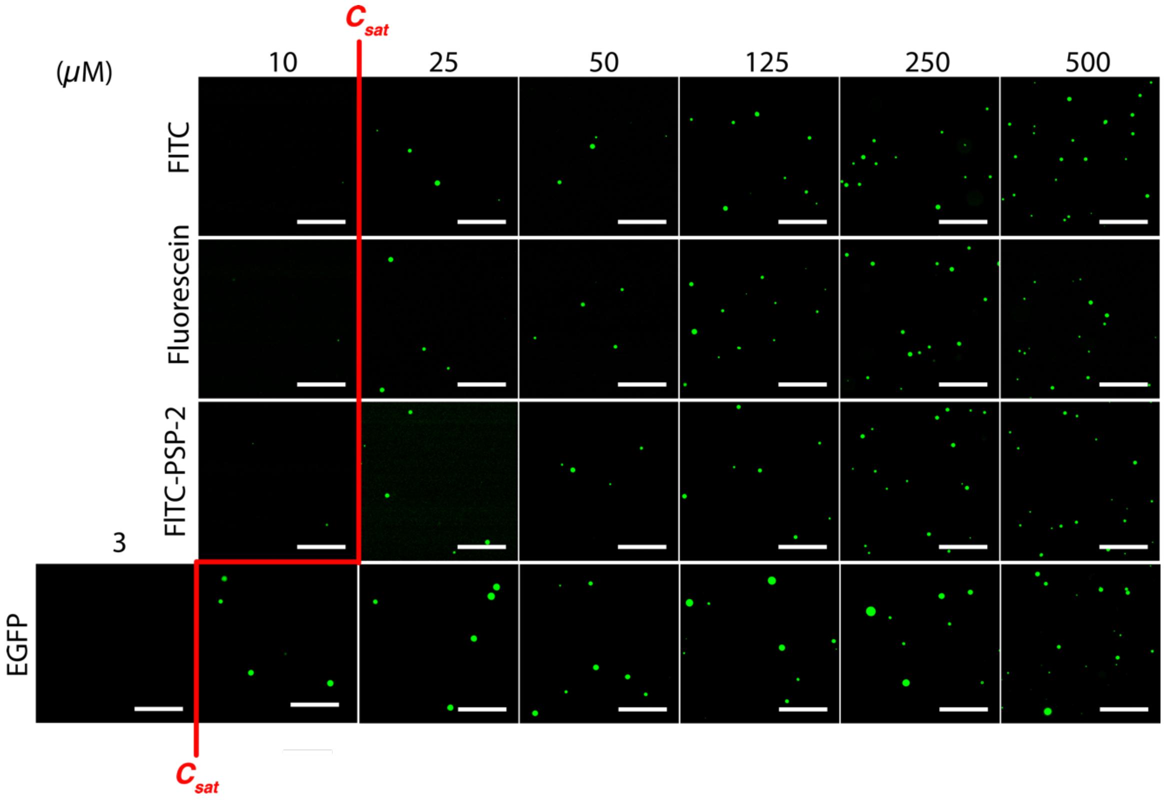

Figure S2: Stability of the different cargo-loaded droplets in the optimum medium at different time points. Confocal images of cargo-loaded PSP-2 self-coacervates in optimum media for FITC, fluorescein, FITC-labeled peptides, or EGFP protein at different dilution states (10 µMM,25 uM,50 uM,100 uM,250 uM and 500 uM). The scale bar is 20 µm.

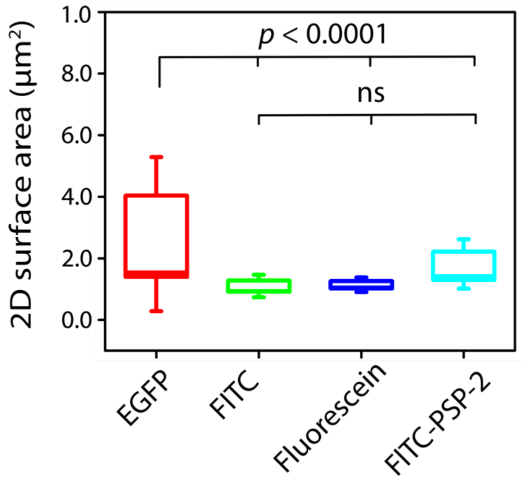

Figure S3: Size of PSP-2 condensates after loading cargo. Sizes of the cargo-loaded self-coacervates in Optimum media after 4 hours of incubation were quantified based on the 2D

bioRxiv preprint doi: https://doi.org/10.1101/2025.05.20.655132; this version posted May 23, 2025. The copyright holder for this preprint (which was not certified by peer review) is the author/funder, who has granted bioRxiv a license to display the preprint in perpetuity. It is made available under aCC-BY 4.0 International license.

surface area observed in the images using ImageJ software for EGFP protein, FITC, fluorescein, and FITC-labeled peptides.

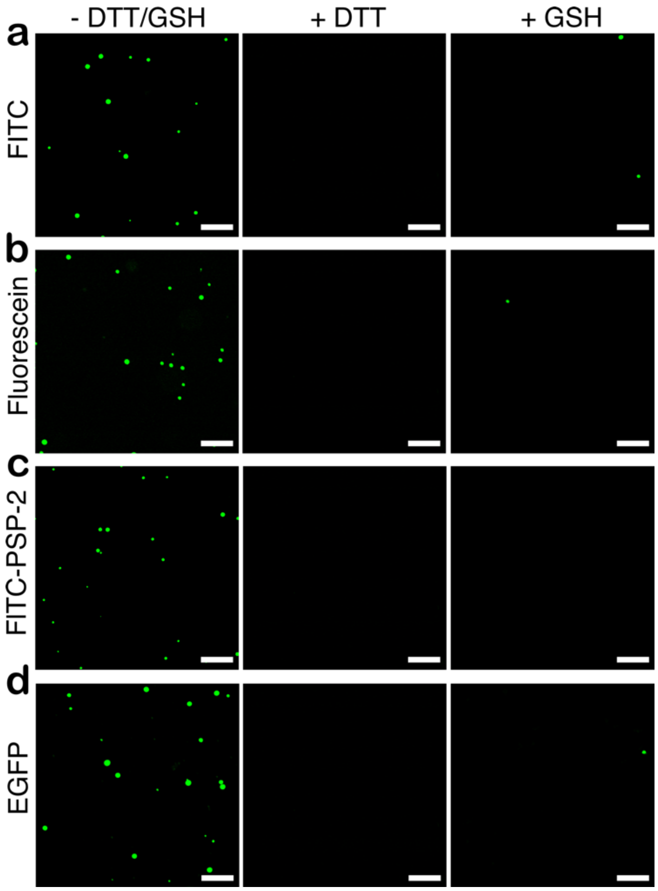

Figure S4: Cargo-laden PSP-2 condensates in Optimum media dissolve in reducing conditions. Confocal microscopy images of different cargo-loaded PSP-2 peptide self-coacervates for FITC (a), fluorescein (b), FITC-labeled peptides (c), and EGFP protein (d) under their respective LLPS conditions. Then, droplets were dissolved using 5mM DTT or GSH. Complete dissolution of condensates was observed after 4 hours incubation with GSH. The scale bar is 20 µm.

bioRxiv preprint doi: https://doi.org/10.1101/2025.05.20.655132; this version posted May 23, 2025. The copyright holder for this preprint (which was not certified by peer review) is the author/funder, who has granted bioRxiv a license to display the preprint in perpetuity. It is made available under aCC-BY 4.0 International license.

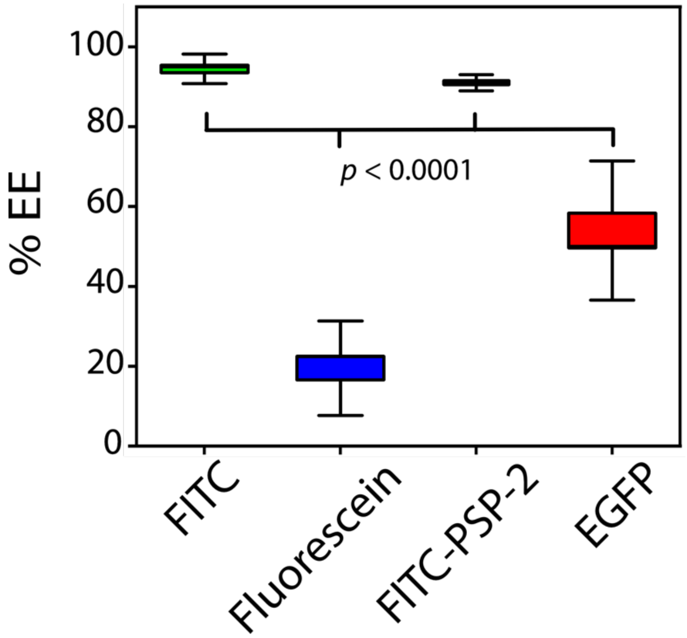

Figure S5: Encapsulation efficiencies of cargo in cell medium. The encapsulation efficiency (%) was assessed for different cargo in Optimum® media. An average of three independent experiments are averaged. Two-way ANOVA between the data sets is shown ($P < 0.0001$).

bioRxiv preprint doi: https://doi.org/10.1101/2025.05.20.655132; this version posted May 23, 2025. The copyright holder for this preprint (which was not certified by peer review) is the author/funder, who has granted bioRxiv a license to display the preprint in perpetuity. It is made available under aCC-BY 4.0 International license.

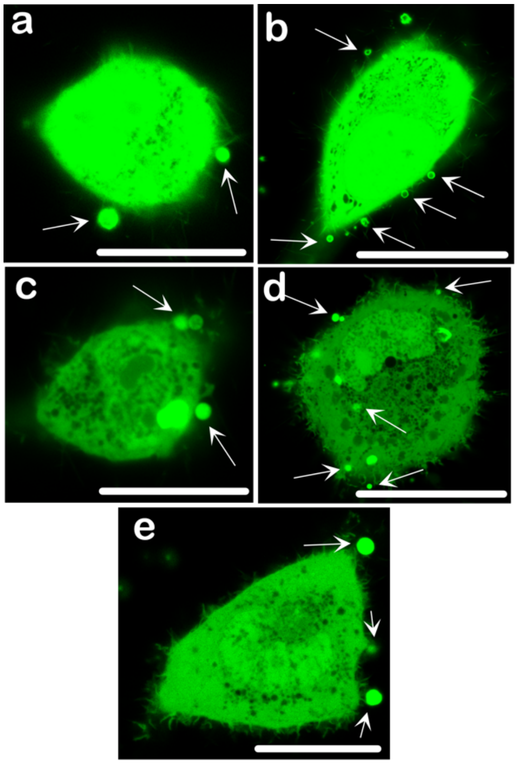

Figure S6: PSP-2 condensates have an affinity for membranes on the extracellular side. Confocal images of EGFP partitioning in HeLa cells show some condensates associated with membrane surfaces on the extracellular side, as indicated by white arrows.

bioRxiv preprint doi: https://doi.org/10.1101/2025.05.20.655132; this version posted May 23, 2025. The copyright holder for this preprint (which was not certified by peer review) is the author/funder, who has granted bioRxiv a license to display the preprint in perpetuity. It is made available under aCC-BY 4.0 International license.

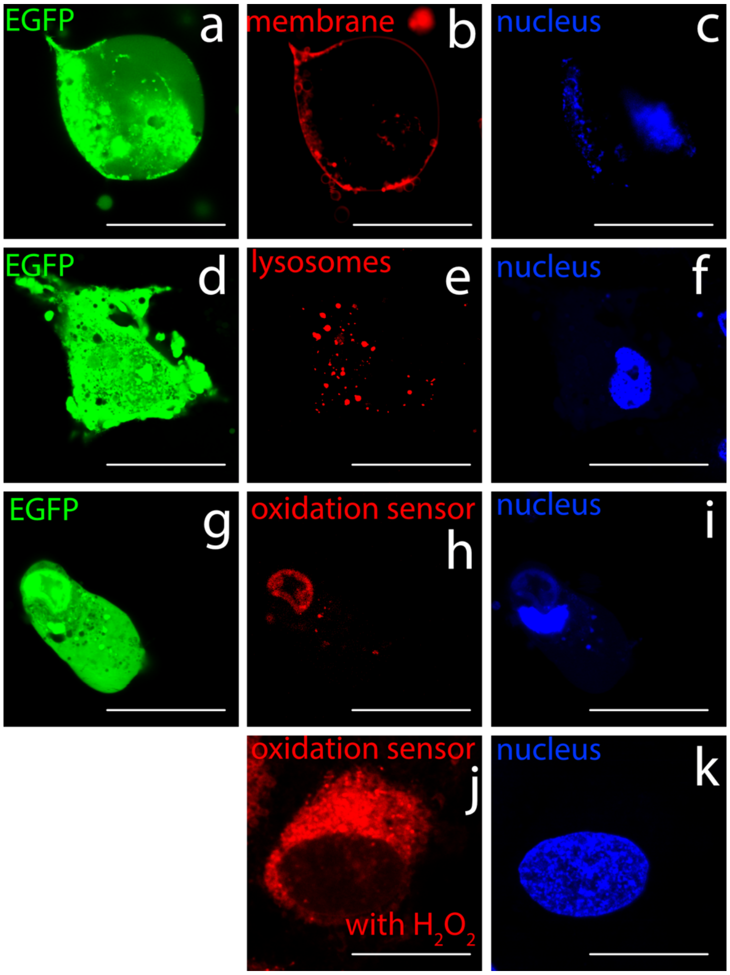

Figure S7: Cellular environment of condensates. Confocal images of EGFP partitioning in HeLa cells. In addition to EGFP (a, d, g) and nucleus (c, f, I, k), the cells were orthogonally stained for membranes (Cellbrite Fix 640 membrane Dye (Biotium)) (b), lysosomes (LysoView 650 (Biotium)) (e), and oxidation sensor (CellROX Deep Red Reagent (Invitrogen)) (j, j).
